# The Impact of Emergency Department Visits on Missed Outpatient Appointments: A Retrospective Study in a Hospital in Southern Italy

**DOI:** 10.3390/nursrep15070229

**Published:** 2025-06-25

**Authors:** Valentina Cerrone, Vincenzo Andretta

**Affiliations:** Department of Medicine, Surgery and Dentistry, University of Salerno, 84084 Fisciano, Italy; valentina.cerrone@sangiovannieruggi.it

**Keywords:** missed appointments, no-show, emergency department, outpatient services, healthcare management

## Abstract

**Background/Objectives**: Missed outpatient appointments contribute to care discontinuity and emergency department (ED) overcrowding. This study investigated the association between missed appointments and ED visits, identifying predictors such as patient characteristics, distance from the hospital, and waiting time. **Methods**: A retrospective analysis was conducted using a dataset of 749,450 scheduled outpatient appointments from adult patients (aged ≥ 18 years). Patients under 18 were excluded. We identified missed appointments and assessed their association with ED visits occurring in the same period. Descriptive statistics, non-parametric tests, and logistic and linear regression models were applied to examine predictors such as age, sex, distance from the hospital, waiting time, the type of service, and medical specialty. **Results**: The overall no-show rate was 3.85%. Among patients with missed appointments, 37.3% also visited the ED. An older age (OR = 1.007; *p* = 0.006) and the male gender (OR = 1.498; *p* < 0.001) were significant predictors of having a scheduled appointment before an ED visit. No significant associations were found for distance or specialty branch. **Conclusions**: Missed appointments are associated with ED utilization. Predictive factors can inform targeted interventions, such as via improved scheduling systems and personalized reminders. Distance alone may not be a barrier, but system-level solutions are needed to address no-show rates and optimize healthcare resource use.

## 1. Introduction

Missed scheduled outpatient appointments, often referred to as “no-shows”, represent a considerable problem in both hospital and outpatient settings. The no-show rate ranges from 10% to 30%, depending on various factors [1,2]. This phenomenon impacts care continuity and contributes to the inefficient use of increasingly limited healthcare resources. Each missed visit can result in wasted time for medical staff and infrastructure while delaying care for other patients [3,4]. Furthermore, missed appointments can worsen patient health outcomes, especially among individuals with chronic conditions, increasing the risk of complications or emergency interventions [5].

The phenomenon is multifactorial. Demographic, socioeconomic, and clinical elements influence adherence to appointments. Among the most cited predictors are age, gender, socioeconomic status, comorbidities, and the complexity of care [6,7]. Recent studies have highlighted that open-access scheduling models can reduce no-show rates [8] and telehealth models are associated with improved appointment adherence compared to traditional in-person visits [9]. Increased no-show frequencies in primary care have also been linked to higher emergency department utilization [10]. Additionally, predictors such as forgetfulness and work commitments have been identified in various outpatient contexts [11]. Less frequently studied is the role of emergency department (ED) visits. Some evidence suggests that frequent ED users have higher no-show rates, potentially due to logistical challenges, mobility issues, or the perception that urgent care resolves their health issues [4].

Moreover, the ED can serve as an alternative for patients who struggle to attend scheduled appointments, preferring immediate care over delayed access. This behavior risks perpetuating a cycle in which the misuse of emergency services exacerbates the outpatient-care no-show problem [12].

Given the incidence of the problem and its impact on healthcare management and service quality, research is needed to explore the specific role of ED access as a potential predictor of missed outpatient appointments. By addressing this knowledge gap, healthcare providers and policy-makers can implement evidence-based strategies to reduce no-show rates and optimize resource allocation.

The main objective of this study was to evaluate the association between ED visits and missed scheduled outpatient appointments in a hospital in the Campania Region. Specifically, we aimed to

1. Measure the no-show rate among a representative patient population.
2. Quantify the frequency of ED visits and examine their relationship with appointment attendance.
3. Identify the key predictors of missed appointments, considering demographic, socioeconomic, clinical, and logistical variables.

## 2. Materials and Methods

### 2.1. Study Design

We conducted a retrospective cohort study in accordance with the STROBE (Strengthening the Reporting of Observational Studies in Epidemiology) guidelines [13]. The aim was to assess the association between emergency department (ED) visits and missed scheduled outpatient appointments. For confidentiality and generalizability purposes, affiliated facilities within the hospital network are referred to as Site A, Site B, etc. instead of using their actual geographic names.

### 2.2. Setting

The study was carried out at a University Hospital in the Campania Region, Southern Italy. We analyzed data from all scheduled outpatient appointments between 1 January 2022 and 30 June 2024. ED visit data for the same patients were collected from 1 July 2021 to 31 December 2023.

### 2.3. Data Sources and Variables

Data were extracted from the hospital’s electronic health record systems: the regional CUP (Unified Booking System) and the internal AREAS operating system. The following information was collected:

- Demographics: age, gender;
- Clinical data: comorbidities, type of outpatient service;
- Logistical data: distance from the patient’s residence to the hospital;
- Missed appointments: total number, waiting time;
- ED access data: reason for visit and timing in relation to the outpatient appointment.

The analysis focused on adult patients (aged 18 years and above) who had had at least one scheduled outpatient appointment in 2022. Missed appointments were identified and linked with emergency department visits occurring in the same timeframe.

Non-urgent ED visits were considered as they may reflect health-seeking behaviors driven by accessibility, perceptions of urgency, or dissatisfaction with routine outpatient care, potentially linked to future non-attendance.

Data preprocessing included removal of duplicate records, correction of entry errors, and management of missing data.

### 2.4. Inclusion and Exclusion Criteria

- Inclusion: adult patients (>18 years) who had missed at least one outpatient appointment between 1 January 2022 and 30 June 2024.
- Exclusion: pediatric patients and individuals without any scheduled appointments during the study period.

An ED visit was defined as any emergency access occurring between the booking date and the scheduled date of the outpatient appointment. The type of outpatient service and the time interval between the ED visit and the appointment were also recorded.

Pediatric patients and those residing more than 150 km from the hospital were excluded to ensure a homogeneous population and minimize confounding related to travel or pediatric-specific care pathways. This may limit generalizability to rural or underserved groups.

### 2.5. Statistical Analysis

Descriptive statistics were used to analyze the variables. For categorical variables, absolute and relative frequencies (*n*, %) were calculated. For continuous variables, means and standard deviations (SDs) were reported for normally distributed data; medians and interquartile ranges (IQRs) were used otherwise. Comparisons between patients who attended and missed their appointments were performed using the t-test for continuous variables and the chi-square test for categorical variables. Logistic and linear regression models were used to

1. Identify predictors of missed outpatient appointments;
2. Analyze the relationship between missed appointments and ED visits.

Mixed-effects models and survival analysis were considered; however, logistic and linear models were selected for their interpretability and alignment with the available cross-sectional structure of the data. Variable selection was based on a priori relevance from the literature and data availability. Multicollinearity among predictors was assessed using Variance Inflation Factor (VIF) statistics, with no significant issues detected. Missing data were handled using listwise deletion after confirming that missingness was below 5% for all key variables. Statistical analyses were performed using Stata version 18 (StataCorp, College Station, TX, USA, 2023) [14]. A completed STROBE checklist is provided as Supplementary Table S1.

## 3. Results

A total of 10,754 outpatient services linked to ED access were analyzed. After applying inclusion and exclusion criteria, 2191 patients remained in the final study sample. Patients under 18 years of age (*n* = 36) and those residing more than 150 km from the hospital (*n* = 71) were excluded as geographic outliers. Only non-urgent ED accesses (white codes) were included, and only those whose ED outcome indicated outpatient referral or patient departure prior to medical evaluation or discharge documentation.

### 3.1. Descriptive Statistics

The mean age of the participants was 55.9 years (SD = 19.05), ranging from 18 to 98 years. The age distribution was symmetric, with a median of 57 years and interquartile range from 41 to 71 years. The gender distribution was balanced: 49.3% female (*n* = 1089) and 50.7% male (*n* = 1119). The mean distance from the patient’s residence to the hospital was 14.6 km (SD = 46.6 km); excluding outliers, the mean was 11.2 km (SD = 16.4 km), ranging from 0 to 136 km. Most patients resided in the main urban area (41.02%), followed by two nearby municipalities, referred to here as Site A (7.50%) and Site B (7.23%), respectively.

The most frequent services were diagnostic and specialist visits:

- Electrocardiogram (2.83%);
- Surgical consultations (3.55%);
- Gastroenterology consultations (3.82%): The main outpatient branches included surgery, gastroenterology, orthopedics, and urology.

**Figure 1:**
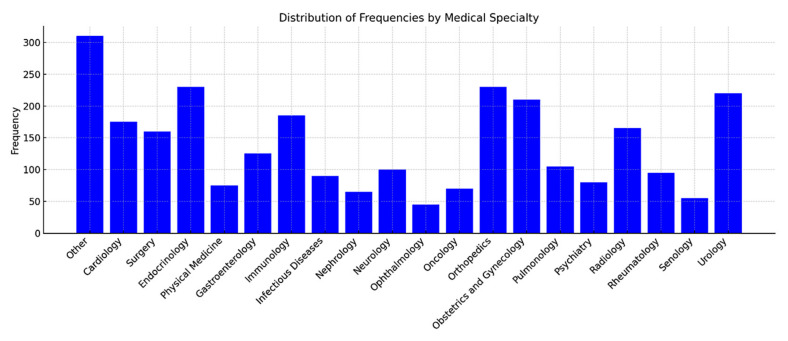
Distribution of frequencies by medical specialty.

Less represented services were grouped under “Other”. The main branches of outpatient healthcare services are represented in the following figure (Figure 1).

### 3.2. Bivariate Associations

Among the 749,450 scheduled outpatient appointments, 28,850 were missed (3.85%). Consistent with our hypothesis, missed appointments were not randomly distributed but showed associations with specific patterns of ED utilization. During the observation period (1 January 2022–30 June 2024), 190,521 ED visits were recorded, representing 25.42% of all appointments. Of the 28,850 missed appointments (Table 1), 10,754 (37.3%) were linked to patients with at least one ED visit; the remaining 62.7% (*n* = 18,096) involved patients without ED access.

### 3.3. Multivariate Analysis

A logistic regression analysis was conducted to identify predictors of having a scheduled appointment before or after an ED visit (Table 2). To test our main hypothesis, we conducted a logistic regression to assess whether specific patient characteristics predicted the likelihood of having a scheduled outpatient appointment prior to an ED visit. Variables included age, gender, travel distance, the type of outpatient clinic, and medical specialty. Older age increased the likelihood of having a scheduled appointment before ED access (OR = 1.007; 95% CI: 1.002–1.012; *p* = 0.006). The male gender also significantly increased the odds (OR = 1.498; 95% CI: 1.244–1.804; *p* < 0.001).

The complexity of service was inversely associated with pre-ED appointments (OR = 0.942; *p* = 0.015), suggesting that more complex services are harder to attend before an ED visit. Distance and specialty branch were not significantly associated (*p* > 0.05).

Each additional year of age increased the likelihood of having an appointment before the ED by 0.7% (OR = 1.007; 95% CI: 1.002–1.012; *p* = 0.006). Older patients were more likely to keep appointments scheduled before accessing the ED.

Men were significantly more likely to keep appointments before the ED than women, with an increase of 49.8% (OR = 1.498; 95% CI: 1.244–1.804; *p* < 0.001).

**Figure 2:**
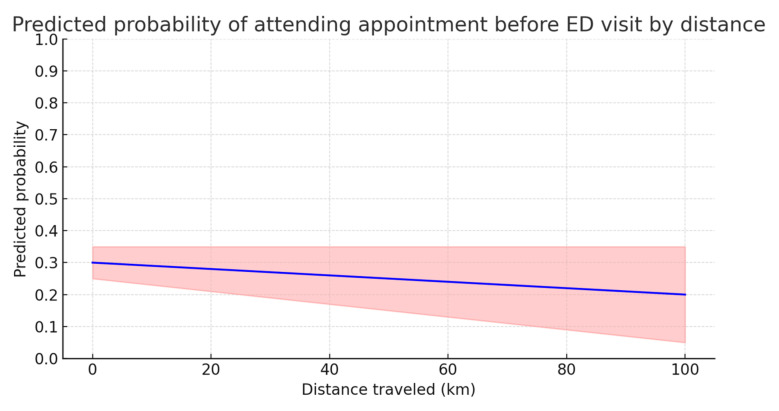
Predicted probability of keeping a scheduled appointment before the emergency department (ED) as a function of distance traveled (in kilometers). The blue line represents the estimated predicted probability while the red shaded area corresponds to the 95% confidence interval.

The complexity of the practice was inversely related to the likelihood of keeping appointments before the ED (OR = 0.942; 95% CI: 0.897–0.989; *p* = 0.015), suggesting that more complex services may make it more difficult to keep scheduled times. The distance traveled and branch of service did not show significant associations with the likelihood of having an appointment before the ED (*p* > 0.05) (Figure 2).

The figure shows that the predicted probability of keeping an appointment before the ED tends to decrease slightly as the distance traveled increases. However, the slope of the line is minimal, suggesting that the effect of distance is not significant. Furthermore, the confidence interval, which widens with distance, indicates greater uncertainty in the estimates for large distances. However, the distance traveled is not a strong determinant of the probability of keeping appointments, although it may have a marginal effect in some contexts.

### 3.4. Group Comparison

**Figure 3:**
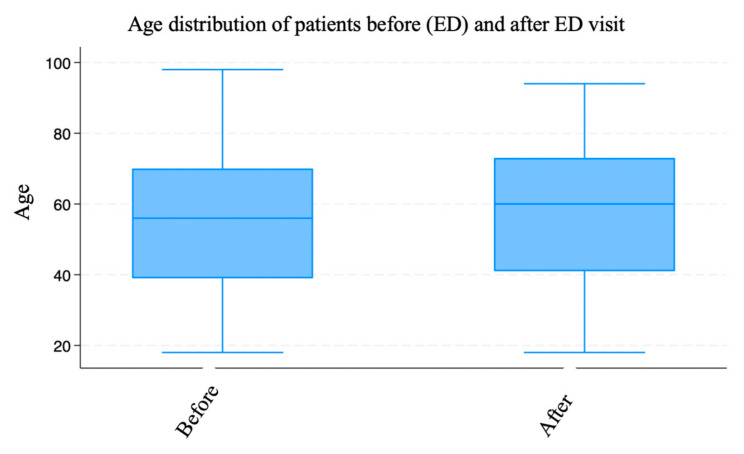
Age distribution of patients divided into those who had scheduled appointments before the emergency department (ED) and those who had them after their ED visit.

Participants with appointments before ED visits were older (mean = 57.45 years, SD = 18.98) than those with appointments after ED visits (mean = 55.14 years, SD = 19.08), with a statistically significant difference (*p* = 0.0095) (Figure 3).

The box plot illustrates the age distribution of patients divided into two groups: those with scheduled appointments before the emergency department (ED) and those with scheduled appointments after the ED visit. Both groups had similar distributions, with the age ranging from 18 to approximately 98 years. However, there was a slight difference in the position of the median. The difference in mean age between the two groups suggested that older patients were more likely to have a scheduled appointment before their ED visits. This could have been due to a greater awareness of the need for scheduled care among older patients or more careful management of their appointments by the healthcare system. In contrast, younger patients appeared to be more likely to visit the ED without a prior scheduled appointment. The graph visually supports the interpretation of the t-test results, showing that although the difference between the groups was relatively small, it was statistically significant (*p* = 0.0095).

### 3.5. Waiting Time and Time Between Appointment and ED Visit

A logistic model was constructed and used to analyze the relationship between waiting time and the time between service and access to the emergency department. The following variables were included: age, sex, distance traveled, and waiting time for the service. The model was significant overall (Prob > χ^2^ = 0.0001), with a Pseudo R^2^ of 0.0123, indicating that although the model explained a relatively small portion of the variability in the time between service and the ED, some factors had a statistically significant impact (Table 3). While the explanatory power of the model is modest, this is not uncommon in health service research involving complex behavioral outcomes such as appointment adherence, where unmeasured social and psychological factors may play a significant role.

Among the factors examined, the waiting time for the outpatient service was a significant predictor of the time between the service and access to the ED. In particular, the model showed that an increase in the waiting time for a service was associated with an increase in the time between the service and access to the emergency department. More precisely, for each additional day of waiting, the time to access the ER increased on average by approximately 0.5% (β = 0.0049, *p* < 0.001). This result suggests that services with longer waiting times are linked to less urgent planning and more oriented towards respecting a scheduled order without immediate recourse to emergency services.

Gender was another significant factor in determining the relationship between waiting time and access to the ED. Men were more likely to respect a scheduled appointment before accessing the ED than women, with a significant increase in probability (β = 0.2875, *p* = 0.010). This could indicate a difference in habits or perception of the need for urgent care between men and women.

In contrast, age and distance traveled were not significant factors for the time between service and access to the ED (*p* > 0.05). These results suggest that in the context analyzed, distance traveled and age do not significantly affect the time between service and access to the ED, suggesting a certain uniformity in the organization and access to health services regardless of the age group or geographical distance.

### 3.6. Service Type and Travel Distance

Significant differences in travel distance were found across medical specialties. The longest travel distances were associated with

- Infectious diseases (*p* < 0.001);
- Radiology, oncology, and cardiology, which had longer distances compared to orthopedics and breast care.

### 3.7. Age and Distance Correlation

A weak but significant negative correlation between age and travel distance was observed (rho = −0.0699; *p* = 0.001), indicating that younger patients tended to travel farther. Gender and specialty branch were significantly associated (*p* < 0.001): infectious diseases were predominantly male (72.73%) while breast care was almost exclusively female (97.22%).

## 4. Discussion

In this study, we analyzed the impact of emergency department visits on missed scheduled healthcare appointments in a hospital facility in the Campania Region. The results showed that demographic variables such as age and gender, as well as the type of clinic and its complexity, significantly influence the probability of having an appointment before or after accessing the ED.

These findings confirmed that an older age and male gender were associated with a higher likelihood of attending scheduled appointments before ED access, in line with our multivariate results. The association between waiting time and appointment adherence appears complex and, at times, counterintuitive. While some studies report that longer delays increase the risk of missed appointments, our findings suggest that patients scheduled for specialized follow-ups—typically associated with longer intervals—may perceive these visits as more important and therefore prioritize them. Conversely, extended waiting times could also drive patients to seek quicker alternatives such as the ED, especially if they perceive outpatient care as inaccessible or inefficient. This dual effect underlines the need to better integrate emergency and scheduled services and to reduce waiting times where possible [3,8,9].

Another interesting result is the relationship between the complexity of the clinic and the probability of having an appointment before the ED. Patients requiring more complex services (such as oncology and cardiology) are less likely to keep appointments. This could be due to a greater difficulty in organizing and keeping appointments for complex treatments, in addition to the possibility that patients, having already received urgent treatment, perceive the need to keep a scheduled outpatient appointment as less urgent. Our result is in line with similar studies that have shown that patients with complex diseases are more likely to rely on emergency services rather than scheduled appointments [7,12,15].

Another aspect that deserved attention was the distance traveled by patients to access healthcare services. Although no significant relationship between distance and non-attendance emerged, the concentration of patients in municipalities near the main hospital (e.g., Site A, Site B) suggests that distance was not a significant obstacle in this specific case. Similar studies, such as those by Sun and Marbouh [1,6], have instead found that distance is one of the main factors associated with non-access to outpatient services. However, the results of our study were likely influenced by the limited geographical context and the ease of access to nearby affiliated facilities.

Several studies have emphasized structural and logistical factors contributing to missed outpatient appointments and emergency department (ED) overcrowding. For example, non-urgent ED visits and staff shortages are recognized as key drivers of crowding, limiting the system’s ability to manage access effectively. Suggested interventions include observation units and better hospital bed allocation to reduce delays and redirect inappropriate visits [15]. Furthermore, geographic distance remains a critical barrier to access. A recent study demonstrated that patients living farther from the healthcare facility were significantly more likely to miss radiology appointments, confirming the role of spatial inequities in healthcare utilization [16]. These findings reinforce our results, highlighting that distance and system-level inefficiencies must be addressed to improve continuity of care and resource planning. Although the pseudo R^2^ values were modest, this is typical in behavioral research involving health service use, and the models remain informative.

An important finding of our study was the prevalence of 3.85% of missed appointments, which may seem low but still represented a considerable loss in terms of health resources. This relatively low rate may be explained by centralized scheduling practices, reminder systems, and the high accessibility of outpatient services in the area. According to Coppa et al. [16], each missed appointment leads to a significant waste of resources and a reduction in the effectiveness of care. This phenomenon is even more relevant in health emergency contexts, where resources are limited, and can exacerbate the pressure on hospitals and emergency rooms.

The findings suggest that while ED visits are associated with missed outpatient appointments, the impact is nuanced and influenced by patient demographics and clinical complexity. Some patients may prioritize emergency care due to acute needs or the perceived inefficacy of scheduled care. Gender also emerged as a relevant factor, with men more likely to attend pre-ED appointments. Although distance was not a significant factor in this study, its relevance in rural or underserved areas remains supported by the broader literature [4]. The integration of ED and outpatient services is crucial for reducing inefficiencies. Reducing waiting times and improving access to outpatient care may prevent unnecessary ED utilization. Coordinating emergency and scheduled care could reduce no-shows and improve healthcare system efficiency [17,18].

### 4.1. Study Limitations

Despite its strengths, the study has several limitations. Selection bias may have arisen from the exclusion of pediatric patients and those living more than 150 km from the hospital, potentially limiting the sample’s representativeness. Although adjustments were made for variables such as age, gender, and distance, unmeasured confounders—like illness severity or educational level—may still have influenced ED visits and appointment adherence.

Measurement bias may have occurred due to reliance on electronic health records, which can inconsistently capture reasons for ED visits or the perceived complexity of care. To address this, data were preprocessed to correct errors, remove duplicates, and manage missing values.

Additionally, the dataset lacked key variables such as socioeconomic status (e.g., income, education), comorbidities, and clinical severity—factors that could act as confounders influencing both outcomes. Future studies should include these variables to enhance explanatory power and minimize residual bias.

### 4.2. Policy Implications

Regional policies, such as the Campania Region’s 2024–2025 health plan, emphasize reducing missed appointments and non-urgent ED visits [19]. The plan mandates healthcare providers to increase outpatient capacity and coordinate better with EDs. This aligns with our findings and underlines the importance of systemic strategies.

Improving outpatient availability and timeliness, patient education, and reminder systems can lower the reliance on emergency services for non-urgent conditions. Programs that address logistical barriers, such as transport support for patients in remote areas, may further enhance adherence to scheduled care.

### 4.3. Generalizability

This study was conducted in a single tertiary hospital in the Campania Region, which may limit generalizability to other healthcare contexts. While the findings reflect a large and diverse outpatient population, further multicenter studies—including rural and urban comparisons—are needed to validate these results in different organizational settings. The integration of regional and national datasets could also support broader policy recommendations.

### 4.4. Strengths and Implications for Practice

This study had several strengths. It was based on a large dataset of over 700,000 outpatient appointments, allowing for robust statistical analyses and reliable estimates of missed appointment prevalence. The use of logistic and linear regression models enhanced the interpretation of predictors associated with follow-up adherence and emergency department (ED) visits. Importantly, the study integrated geographic data (distance from hospital) and sociodemographic characteristics, offering a multidimensional view of healthcare access. The findings have direct practical implications: healthcare administrators should consider redesigning care pathways to identify high-risk patients and implement targeted reminder systems or telehealth alternatives. Moreover, policy-makers should address structural barriers, including service decentralization and workforce allocation, to reduce non-adherence and ED overcrowding.

## 5. Conclusions

This study explored the impact of emergency department (ED) visits on missed scheduled outpatient appointments, aiming to identify the main factors associated with this phenomenon. The findings confirmed that no-show behavior is influenced by a combination of demographic, clinical, and logistical factors, including age, gender, distance from the hospital, and service complexity. In particular, older patients and males were more likely to attend appointments prior to an ED visit, suggesting that age- and gender-related health behaviors affect access to care. The data also indicated that complex services are associated with lower appointment adherence, possibly due to difficulties in scheduling or decreased urgency after receiving emergency care. Importantly, this study supported the notion that the integration of outpatient and emergency services could help reduce system inefficiencies. Coordinated care pathways may improve patient flow, reduce missed appointments, and lower inappropriate ED usage. These improvements could lead to better health outcomes and the more efficient use of healthcare resources. The findings align with regional health policies such as the Campania Region’s 2024–2025 plan, which emphasizes increasing outpatient service availability and reducing the number of unnecessary ED visits. By implementing targeted strategies—such as improving access, decreasing wait times, and enhancing coordination—health systems may effectively address the no-show problem and improve overall care delivery. Future research should include multicenter and longitudinal designs, integrating detailed socioeconomic and clinical variables, to validate these findings and enhance their generalizability across healthcare systems.

## Figures and Tables

**Table 1:** Proportions of outpatient services compared to emergency room visits.

State	Frequency	Percentage (%)
With ED access	10,754	37.3%
Without ED access	18,096	62.7%
Total	28,850	100%

**Table 2:** Logistic regression results—predictors of attending an appointment before ED visit.

Variable	Odds Ratio (OR)	95% Confidence Interval	*p*-Value
Age	1.007	1.002–1.012	0.006
Sex (male vs. female)	1.498	1.244–1.804	<0.001
Distance traveled	0.997	0.991–1.003	0.386
Type of outpatient clinic	0.942	0.897–0.989	0.015
Medical specialty	0.994	0.980–1.008	0.396

**Table 3:** Linear regression results—predictors of time interval between outpatient appointment and ED visit.

Variable	Coefficient (β)	Standard Error	*p*-Value	95% Confidence Interval
Waiting time	0.0049	0.0013	<0.001	(0.0023–0.0074)
Age	0.0012	0.0029	0.697	(−0.0046–0.0069)
Sex (male vs. female)	0.2875	0.1111	0.010	(0.0698–0.5053)
Distance traveled	−0.0034	0.0034	0.318	(−0.0099–0.0032)

## Data Availability

The data analyzed in this study are not publicly available due to privacy restrictions and institutional data use agreements. Requests for access to aggregated or anonymized data may be considered by the corresponding author upon reasonable request and with appropriate approvals.

## Supplementary Materials

The following supporting information can be downloaded at: https://www.mdpi.com/article/10.3390/nursrep15070229/s1, Table S1: STROBE Checklist for Observational Studies.

## Public Involvement Statement

There was no public involvement in any aspect of this research.

## Guidelines and Standards Statement

This manuscript was drafted in accordance with the STROBE (Strengthening the Reporting of Observational Studies in Epidemiology) guidelines for observational studies [13].

## Abbreviations

The following abbreviations have been used in this manuscript:

ED	Emergency Department
OR	Odds Ratio
CI	Confidence Interval
SD	Standard Deviation
GDPR	General Data Protection Regulation
STROBE	Strengthening the Reporting of Observational Studies in Epidemiology
