# Supplementary material for: The Impact of Emergency Department Visits on Missed Outpatient Appointments: A Retrospective Study in a Hospital in Southern Italy
Source: Nurs Rep. 2025 Jun 25;15(7):229. doi: 10.3390/nursrep15070229 (PMC12300468; doi:10.3390/nursrep15070229)
Supplement: Supplementary file 1 [file nursrep-15-00229-s001.zip › nursrep-3651177-supplementary.pdf]

nursing reports

MDPI

Supplementary Table S1: STROBE Checklist for Observational Studies

Item	Recommendation	Location in Manuscript
**1. Title and abstract**	Indicate the study's design with a commonly used term in the title or the abstract.	Title and Abstract
**2. Background/rationale**	Explain the scientific background and rationale for the investigation being reported.	1. Introduction
**3. Objectives**	State specific objectives, including any prespecified hypotheses.	1. Introduction
**4. Study design**	Present key elements of study design early in the paper.	2.1 Study Design
**5. Setting**	Describe the setting, locations, and relevant dates, including periods of recruitment and data collection.	2.1 Study Design
**6. Participants**	Give the eligibility criteria, and the sources and methods of selection of participants.	2.2 Study Population
**7. Variables**	Clearly define all outcomes, exposures, predictors, potential confounders, and effect modifiers.	2.3 Variables and Data Sources
**8. Data sources/measurement**	For each variable of interest, give sources of data and details of methods of assessment (measurement).	2.3 Variables and Data Sources
**9. Bias**	Describe any efforts to address potential sources of bias.	4.5 Strengths and Limitations
**10. Study size**	Explain how the study size was arrived at.	2.2 Study Population
**11. Quantitative variables**	Explain how quantitative variables were handled in the analyses.	2.4 Statistical Analysis
**12. Statistical methods**	Describe all statistical methods, including those	2.4 Statistical Analysis

	used to control for confounding.	
**13. Participants (Results)**	Report numbers of individuals at each stage of study.	3.1 Characteristics of the Study Population
**14. Descriptive data**	Give characteristics of study participants and information on exposures and potential confounders.	3.1 Characteristics of the Study Population
**15. Outcome data**	Report numbers of outcome events or summary measures.	3.2 Appointment Attendance and Waiting Times
**16. Main results**	Give unadjusted estimates and, if applicable, confounder-adjusted estimates and their precision.	3.4 Multivariable Regression Models
**17. Other analyses**	Report other analyses done, e.g., subgroup and sensitivity analyses.	3.5 Additional Analyses
**18. Key results**	Summarize key results with reference to study objectives.	4.1 Summary of Main Findings
**19. Limitations**	Discuss limitations of the study, taking into account sources of potential bias or imprecision.	4.5 Strengths and Limitations
**20. Interpretation**	Give a cautious overall interpretation of results considering objectives, limitations, multiplicity of analyses, results from similar studies, and other relevant evidence.	4.3 Interpretation of the Results
**21. Generalisability**	Discuss the generalisability (external validity) of the study results.	4.6 Generalizability
**22. Funding**	Give the source of funding and the role of the funders.	Declarations – Funding
